# Correlation study between serum neuro-specific enolase and gastric and colorectal cancers

**DOI:** 10.1097/MD.0000000000019796

**Published:** 2020-04-17

**Authors:** Hai Luo, Kexin Shen, Hongyan Sun, Ruiqi Li, Zeming Wang, Zhongshi Xie

**Affiliations:** China-Japan Union Hospital of Jilin University, Changchun, Jilin Province, China.

**Keywords:** colorectal cancers, diagnosis, gastric cancers, neuron-specific enolase

## Abstract

This study investigated the diagnostic value of preoperative serum neuro-specific enolase (NSE) in gastric cancer (GC) and colorectal cancer (CRC), and the diagnostic viability of combined serum NSE, carcinoembryonic antigen (CEA), cancer antigen (CA)19-9, and CA242.

Patients with GC and CRC, and a healthy control group (n = 666 and 266, respectively) were compared with regard to NSE, CEA, CA19-9, and CA242 serum levels. NSE was analyzed for associations with clinicopathological parameters. To estimate the diagnostic potential of NSE, a receiver operating characteristic curve was constructed and the area under the curve (AUCs) was calculated for different patient subgroups.

The median serum NSE level of the tumor group (20.925 ng/mL) was significantly higher than that of the control (15.190 ng/mL). Serum NSE was associated with pathological tumor-node-metastasis staging, lymph node metastasis, distant metastasis, vascular invasion, and nerve infiltration. The area under the receiver operating characteristic curve (AUC) for NSE in GC and CRC (0.769) was higher than for the other 3 markers (0.571–0.680). The AUC of the combined markers was higher than for any of the markers individually (0.778–0.810).

The AUC for NSE alone suggests it may be an independent tumor marker, and useful for diagnosis of GC and CRC. However, the AUC for combined NSE, CEA, CA19-9, and CA242 was higher and thus potentially more diagnostic value.

## 1 Introduction

Cancer is an important global health issue that significantly affects patient morbidity and mortality.^[1]^ According to the 2018 GLOBOCAN (Global Cancer Incidence, Mortality, and Prevalence) report, gastric and colorectal cancers (GC and CRC, respectively) account for 15.9% of new cases of cancer and 17.4% of the total number of deaths, which is higher than for other cancers.^[2]^ Because of the increasing incidence of GC and CRC, and to extend patient survival, the World Health Organization has recommended focusing on early detection and follow-up after surgery.^[3]^

The most widely used diagnostic approaches for gastrointestinal tumor are endoscopic, with high sensitivity and specificity for identifying polyps and cancers. Such procedures include electronic gastroscope, colonoscopy, and sigmoidoscopy.^[4]^ However, these tests are invasive and expensive, and patient compliance is poor.^[5]^ Non-invasive and inexpensive methods, such as screening with the fecal occult blood test (FOBT), have lower sensitivity and specificity.^[6,7]^

Studies have implicated tumor-derived exosomes in the genesis and development of CRC, and in invasion and metastasis, and theoretically they could be of diagnostic value.^[8,9]^ However, the practicality in a clinical setting is low, due to the complexity of surgical collection, time and high cost, and possibly low sensitivity and specificity. Therefore, there remains an urgent need to develop simpler, less invasive, and more accurate diagnostic methods for GC and CRC.

In recent years, tumor markers have become commonly used in cancer for early screening and diagnosis, guidance of treatment, evaluation of curative effect, monitoring of recurrence and metastasis, and judgment of prognosis and survival.^[10]^ Especially, carcinoembryonic antigen (CEA) has shown great value for differential diagnosis, disease monitoring, and evaluation of efficacy of treatment of malignant tumors. In particular for the present study concerning CRC, serum CEA is associated with prognosis and is an indicator of treatment effect and recurrence.^[11,12]^ Other serum markers such as cancer antigen (CA)19-9^[13]^ and CA242^[14]^ have also been used for the diagnosis of GC and CRC and postoperative monitoring of therapeutic effect. Therefore, traditional gastrointestinal tumor markers are feasible and effective for the screening, diagnosis, and postoperative monitoring of gastrointestinal tumor recurrence.

Neuron-specific enolase (NSE) is a cell-specific isoenzyme of the glycolytic enzyme enolase that was first found in extracts of brain tissue.^[15]^ NSE as a serum marker has been widely used in the clinical diagnosis of various benign diseases. Serum NSE is directly proportional to brain damage caused by conditions such as cerebral ischemia, and it is an important biological marker of severe brain injury.^[16,17]^ In addition, NSE is a highly specific marker to be used in diagnosis and prognosis of malignant tumors.^[18]^ Serum NSE has been associated with melanoma, seminoma, renal cell carcinoma, Merkel cell tumor, carcinoid tumors, dysgerminomas and immature teratomas, and malignant pheochromocytoma, especially those arising in small cell lung cancer (SCLC).^[19]^ However, there are few studies regarding the value of NSE in GC and CRC.

This study aimed to the diagnostic value of preoperative serum NSE in GC and CRC, and the diagnostic viability of combined serum NSE, CEA, CA19-9, and CA242.

## 2 Methods

### 2.1 Detection of serum tumor markers

Serum samples were preoperatively collected from each patient. Fasting elbow venous blood was taken between 0600 and 0700 hours on the second day of admission and submitted to the central research office of China-Japan Union Hospital of Jilin University for quantitative marker analysis. Two milliliters of serum from each patient was centrifuged at 3500 × *g* for 5 minutes; the supernatant was added to the corresponding tumor kit for detection. All laboratory tests were conducted in accordance with standard operating procedures. The experiments were performed on a daily basis, because the results are used to guide doctors’ clinical decisions. Based on the manufacturer's instructions from the tumor kits, the serum cut-off values were as follows: NSE, 25.00 ng/mL; CEA, 5.00 ng/mL; CA19-9, 37.00 U/mL; and CA242, 20.00 U/mL, a value higher than the cut-off was considered positive.

### 2.2 Statistical analysis

Correlations between each of the 4 tumor biomarkers in serum and clinicopathological characteristics were analyzed with Pearson chi-squared (χ^2^) test. The serum levels of the 4 tumor biomarkers between the patients and control group were evaluated using the Mann–Whitney *U* test. The Kruskal–Wallis test was employed to compare the serum levels of the 4 markers in patients stratified by tumor site and the healthy controls; pairwise comparisons were performed afterwards with the significance values adjusted by the Bonferroni correction. Correlations among the 4 tumor markers were assessed using Spearman's correlation. Binary logistic regression was applied to evaluate associations between the 4 biomarkers and clinicopathological parameters.

For each tumor biomarker, and the combination of all 4 biomarkers, a receiver operating characteristic (ROC) curve, area under the ROC (AUC), 95% confidence interval (CI), and Youden index (sensitivity + specificity – 1) were calculated. Logistic regression was conducted to analyze the probability of diagnosing GC and CRC by combing the 4 biomarkers. The Hosmer–Lemeshow goodness-of-fit test was used to assess the model.

All of the above statistical analyses were performed using SPSS 25.0 software (SPSS, Chicago, IL). MedCalc V15.2 software was used to perform the *Z* test and compare the AUCs of the combined test with each single biomarker. *P* < .05 was considered statistically significant.

## 3 Results

### 3.1 Patients and samples

The patient group (n = 666; 434 men; aged 27–87 year) comprised those hospitalized from July 2015 to March 2019 at China-Japan Union Hospital of Jilin University, specifically with GC, colon, or rectal cancer (n = 219, 221, and 226, respectively). Serum samples were collected from each patient. Tumor staging was conducted based on the tumor-node-metastasis (TNM) classifications of the American Joint Committee on Cancer Staging (AJCC, eighth edition).

In this study, 266 healthy volunteers (aged 20–75 year) were included as a control group. These volunteers were free from any viral infections or gastrointestinal disease.

For inclusion in this study, none of the patients had received preoperative neoadjuvant chemoradiotherapy; all underwent radical surgery; postoperative pathological examinations confirmed adenocarcinoma; and each patient had complete clinical and pathological data.

### 3.2 Correlations between NSE level and clinicopathological parameters

There were significant correlations between the NSE level and clinicopathological parameters of patients with GC and CRC (Table 1). The NSE levels of patients with T3/T4 stage were significantly higher than that of patients with T1/T2. NSE level significantly correlated with lymph node metastasis, distant metastases, TNM staging, vascular invasion, and nerve infiltration. There were no statistically significant associations with gender, age, or degree of tumor differentiation.

**Table 1:**
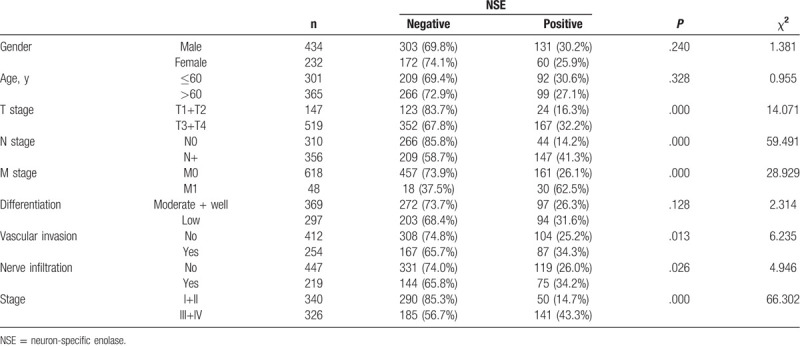
NSE levels by clinicopathological parameter.

### 3.3 Median serum levels of NSE, CEA, CA19-9, and CA242 in healthy controls and pretreatment patients

For analysis, the patients were stratified by tumor site (Table 2). The serum levels of the 4 markers in the GC and CRC patients were significantly higher than that of the control group. Specifically, in patients with GC and CRC (healthy controls), the median levels of the serum biomarkers were as follows: NSE, 20.952 (15.190) ng/mL; CEA, 3.040 (1.785) ng/mL; CA19-9, 11.580 (9.535) U/mL; and CA242, 6.240 (3.755) U/mL.

**Table 2:**
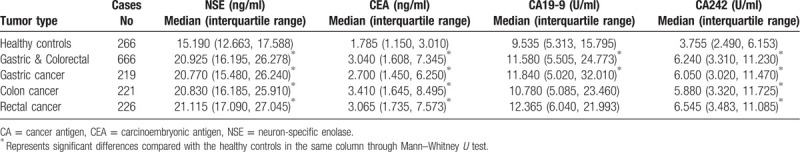
Median serum levels of NSE, CEA, CA19-9, and CA242 in healthy controls and patients prior.

The patients were further stratified as having GC, colon cancer, or rectal cancer. The serum levels of NSE, CEA, and CA242 were significantly higher in each of these patient groups compared with the healthy group. The serum CA19-9 concentration of the GC group was significantly higher than that of the healthy control group. However, the serum CA19-9 concentrations of the colon cancer group, rectal cancer group, and healthy group were comparable.

### 3.4 Binary logistic regression of NSE vs GC and CRC clinicopathological parameters

To further understand the relationship between the NSE with GC and CRC clinic-pathological parameters, binary logistic regression analysis was also performed. Our study indicated that lymph node metastasis, distant metastasis can significantly affect NSE level in GC and CRC patients (Table 3). However, NSE levels were not affected by the T staging, vascular invasion, nerve Infiltration, as well as the pathological tumor-node-metastasis staging.

**Table 3:**
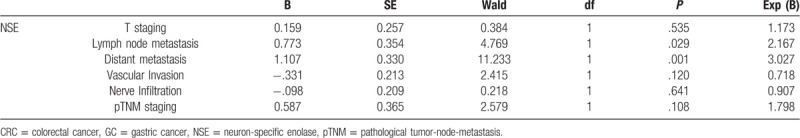
Binary logistic regression analysis of NSE and GC and CRC clinicopathological parameters.

### 3.5 Serum NSE levels in GC and CRC

**Figure 1:**
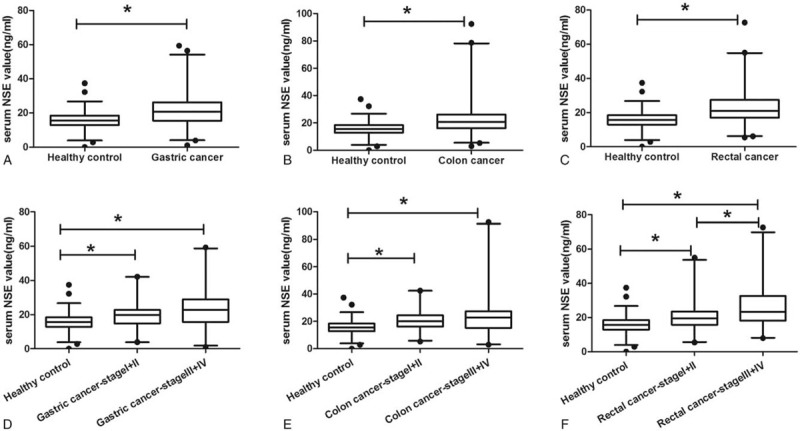
Range of serum NSE levels in gastric and colorectal cancer. Box plot of distribution of NSE in serum in gastric, colon, and rectal cancers and healthy controls. The center line and box represent the median and interquartile ranges, respectively. ∗*P* < .05. NSE = Neuron-specific enolase.

In each of the GC, colon cancer, and rectal cancer groups, the concentrations of NSE were significantly higher relative to the control group (Fig. 1). The specific range (median) of each of the patient groups and control group were as follows: GC, 1.08 to 59.25 ng/mL (20.77 ng/mL); colon cancer, 2.92 to 92.45 ng/mL (20.83 ng/mL); rectal cancer, 5.41 to 72.72 ng/mL (21.11 ng/mL); and control group, 2.84 to 37.40 ng/mL (15.19 ng/mL). Compared with the healthy control group, the serum NSE level was significantly higher in patients with either early or advanced stages (I+II and III+IV, respectively). In patients with rectal cancer, the NSE levels of those at advanced stage were significantly higher than that of patients with early stage.

### 3.6 Associations between tumor markers and clinical stage of disease

**Figure 2:**
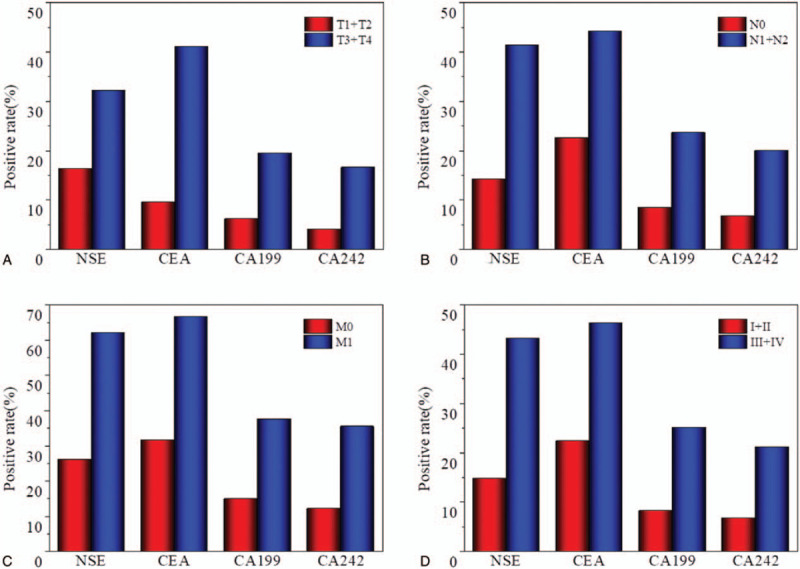
Percentages of patients testing positive for the 4 tumor markers, according to (A)T stage, (B) N stage, (C) M stage, and (D) pTNM stage.

The patient group was divided into 2 subgroups according to early and advanced clinical stages (I+II and III+IV, respectively; Fig. 2). The rates of positivity of each of the 4 tumor markers increased with the clinical stage. Serum levels of NSE were significantly higher in patients with T3+T4 stage relative to those with T1+T2. In addition, the rate of positivity for serum NSE was significantly higher in patients with lymph node metastasis or distant metastasis compared with those without these, respectively.

### 3.7 Logistic regression and ROC curve analyses

**Figure 3:**
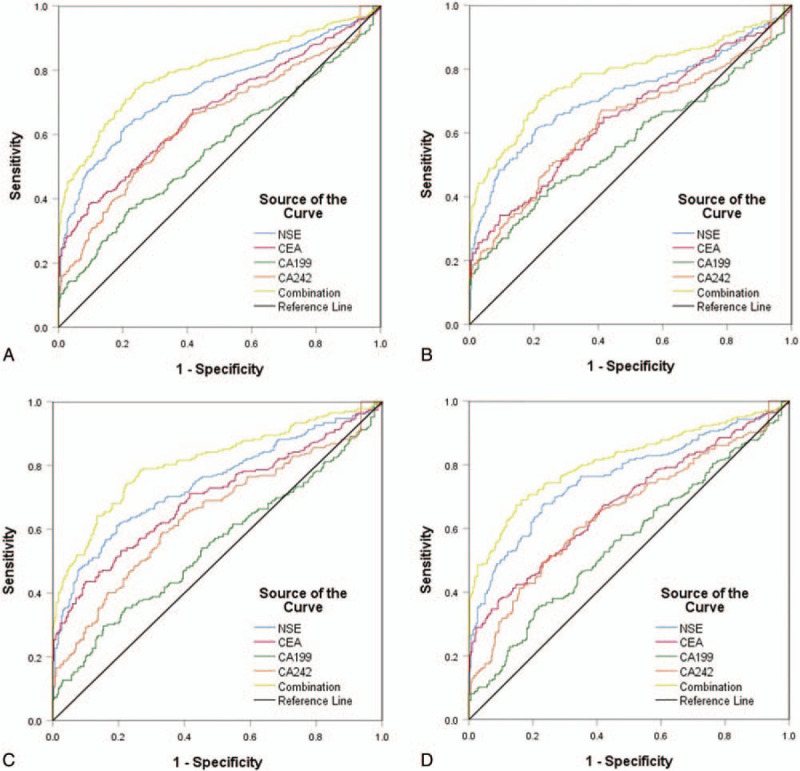
Receiver operating characteristic curves of neuron-specific enolase, carcinoembryonic antigen, CA19-9, and CA242 each alone and in combination in predicting (A) gastric and colorectal cancer, (B) gastric cancer, (C) colon cancer, and (D) rectal cancer. CA = cancer antigen.

For the GC and CRC group, ROC curves were constructed for each of the 4 tumor markers, and the combination of all 4 markers (Fig. 3). For the overall population of 666 patients, the AUCs for each of the markers were as follows (Table 4): NSE, 0.769; CEA, 0.680; CA19-9, 0.571; and CA242, 0.653. The AUC for the combination of all 4 markers was 0.799.

**Table 4:**
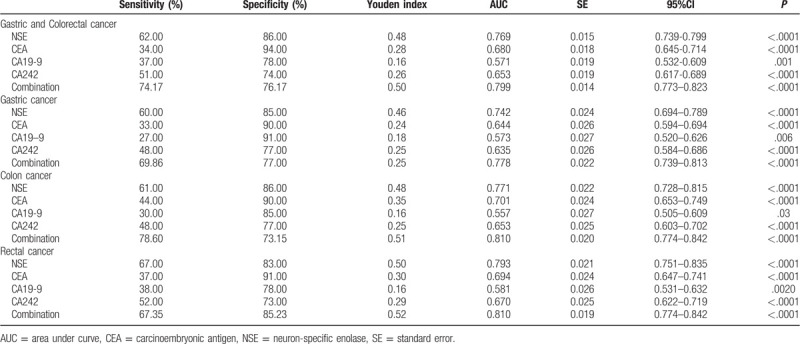
AUCs and the corresponding 95% CIs of combined NSE, CEA, CA19-9, and CA242 (3 different cancers cf. healthy controls).

The Hosmer–Lemeshow test was constructed to verify the appropriateness of the logistic regression model to predict GC and CRC, and the tumor site of CRC. The results were as follows: GC, χ2 = 3.347, *P* = .8511; colon cancer, χ2 = 6.505, *P* = .591; and rectal cancer, χ2 = 14.492, *P* = .070.

The ROC curves were also analyzed for each of the subgroups based on tumor site (GC, colon cancer, and rectal cancer; Fig. 3). For the GC subgroup, the AUCs of the 4 markers NSE, CEA, CA19-9, and CA242, and their combination were: 0.742, 0.644, 0.573, 0.635, and 0.778, respectively. In the colon cancer group, the AUCs of the corresponding markers and their combination were: 0.771, 0.701, 0.557, 0.653, and 0.810. In patients with rectal cancer, the corresponding AUCs were: 0.793, 0.694, 0.581, 0.670, and 0.810. The above data indicated that there was a significant difference in AUCs between the combined test and any single tumor marker in patients with GC and CRC (*P* < .01).

The AUCs of the individual tumor markers and their combination were compared by *Z* test for GC and CRC overall, and the subgroups based on tumor location. For the patient population overall, relative to the combination of all markers, the *Z* values were as follows (all *P* < .01): NSE, 6.105; CEA, 8.157; CA19-9, 12.483 and CA242, 8.676. In the GC subgroup specifically, the corresponding *Z* values were 3.853, 5.592, 7.420, and 5.328, respectively (all *P* < .01). In the colon cancer subgroup, the corresponding *Z* values were 4.391, 5.293, 9.528, and 6.615 (all *P* < .01). In the rectal cancer subgroup, the corresponding *Z* values were 4.216, 6.229, 9.092, and 6.364 (all *P* < .01).

## 4 Discussion

This study investigated whether NSE can serve as a marker for early diagnosis and prognosis in patients with GC and CRC. Serum levels of NSE were compared between 666 patients with GC and CRC and 266 healthy individuals. Associations between serum NSE and clinicopathological parameters were also analyzed. To evaluate the diagnostic ability of NSE, ROC curves were constructed and the AUCs calculated. It was found that the serum NSE levels of the patients were significantly higher than that of the healthy controls. The AUC indicated that NSE has good value for the diagnosis of GC or CRC. Serum NSE levels were associated with pathological tumor-node-metastasis staging, lymph node metastasis, and synchronous distant metastasis in GC and CRC. In addition, the combined detection of the tumor markers NSE, CEA, CA19-9, and CA242 was shown to have great diagnostic significance.

In this study, the positivity rate for NSE in the patients (28.68%) was much higher than that of the healthy individuals (1.88%), and the median serum NSE level of the patients was significantly higher than that of the control. Previous studies have shown that serum NSE levels in patients with malignancies are markedly elevated relative to healthy persons.^[19]^

The relationship between serum NSE levels and GC and CRC has been previously reported, but these results are old and limited.^[20,21]^ Here, we investigated an association between NSE values and clinical staging. The results showed that the serum NSE level did correlate with GC and CRC staging; the serum NSE level of patients at advanced stages (III+IV) was significantly higher than that of patients at early stages (I+II patients). The rates of positivity for NSE in the early and advanced stages were 14.71% and 43.45%, respectively. This suggests that Serum NSE elevation may be an important prognostic factor for GC or CRC.

At the same time, significant correlations were determined between the serum NSE level and the T, N, and M stages. This indicates that serum NSE may reflect the depth of tumor invasion, and correlate with lymph node metastasis and synchronous distant metastasis. Therefore, NSE may be a good indicator for assessing local lymphatic or distant metastasis of GC and CRC. It has been reported previously that the level of NSE correlates with tumor growth and number of metastatic sites.^[15]^

The cause of differences in NSE values between earlier and later clinical stages in GC and CRC is not fully understood. It seems likely that the NSE level is closely related to and may reflect the rate of tumor growth. Enolase is a cytoplasmic enzyme that catalyzes the conversion of 2-phosphoglycerate to phosphoenolpyruvate in the glycolytic pathway. In a setting of tumor growth or inflammation, enolase can be released from the cell to control cell growth, immune tolerance, and allergy.^[22]^ The present results warrant further experiments and follow-ups to confirm that NSE is associated with tumor activity.

Liu et al. showed that elevated CEA before treatment and positive lymph node staging on magnetic resonance imaging were independent risk factors for synchronous distant metastasis in rectal cancer.^[23]^ The combination of both risk factors could indicate patients at high risk and candidates for structured personalized treatment. In the present study, the clinicopathological correlation analysis showed that NSE was significantly associated with vascular invasion and nerve infiltration. This indicates that evaluation of preoperative synchronous metastasis by NSE combined with imaging examination is of great significance. Elevated serum NSE in patients with GC or CRC also may be related to nerve infiltration. Eren et al^[24]^ showed that NSE may upregulate the expression of vascular endothelial growth factor (VEGF) and affect the incidence of lymph node metastasis, promoting neoangiogenesis. However, we know that bevacizumab mainly acts on VEGF. Therefore, bevacizumab may be more effective in the treatment of patients with advanced gastric adenocarcinoma with elevated serum NSE, which requires further experiments and follow-up to demonstrate. Relevant studies concerning this are currently limited but are warranted.

The 666 patients were stratified by primary tumor site, specifically 219, 221, and 226 with GC, colon cancer, and rectal cancer, respectively, and the range of serum NSE levels in each subgroup and control group were compared. The results showed that NSE was a viable marker of diagnosis and prognosis of gastrointestinal tumor at each of the tumor locations. In addition, in patients with rectal cancer, the NSE range of those at advanced stage was significantly higher than that of patients at early stages. Thus, serum NSE values may be a good predictor of rectal cancer staging.

To evaluate the diagnostic ability of NSE and other tumor markers, ROC curves were constructed and the AUCs were calculated. AUC values of ≥0.97, 0.93 to 0.96, 0.75 to 0.92, and <0.75 are respectively considered excellent, very good, good, and deficient or close to random.^[25]^ In the present study, the AUC for NSE in the patient group was 0.769, or good (Table 4). Thus, according to the AUC standard classifications, serum NSE may be an independent tumor marker for GC and CRC. However, the accuracy of NSE alone for diagnosing CRC was not satisfactory, and NSE combined with other frequently-used tumor markers, was investigated, that is, CEA, CA19-9, and CA242. Although these tumor markers have been widely used for diagnosis of various types of cancer, when they are used individually, the results are inconsistent.

The current study found that the sensitivity and specificity of NSE in the diagnosis of GC and CRC were 62.00% and 86.00%, respectively. Compared with the other three tumor markers commonly used, NSE is relatively reliable for the diagnosis of GC and CRC. The sensitivities of NSE in the diagnosis of gastric, colon, and rectal cancer were 60.00%, 61.00%, and 67.00%, and the specificities were 85.00%, 86.00%, and 83.00%. Therefore, it is not difficult to conclude that NSE also has good value for the subgroups based on tumor sites. Similarly, the sensitivity of CEA to GC and CRC was 34.00%, and the specificity was as high as 94.00%. The results are similar to those of McKeown et al,^[26]^ in which the reported sensitivity and specificity of CEA in CRC screening was 36% and 87%. In our study, the sensitivities for CA19-9 and CA 242 in GC and CRC were 37.00% and 51.00%, which suggests that CA 19-9 and CA242 had limited value for the diagnosis of GC and CRC. Therefore, we consider that the diagnostic model combining NSE and CEA may be the simplest and most effective screening method. In this study, the 4 tumor markers (NSE, CEA, CA19-9, and CA242) were combined in a logistic regression model and the diagnostic accuracy was higher, with better sensitivity for GC and CRC.

This study is limited in that the patients were from a single center, insufficient sample size and follow-up is lacking. Therefore, NSE as a prognostic indicator in GC and CRC remains to be clarified. But we believe that the correlation between NSE and GC and CRC will become more clearly in subsequent studies, NSE may be of great value in monitoring recurrence of GC and CRC and selecting adjuvant therapy in the foreseeable future.

## 5 Conclusion

This study found that serum NSE may be an independent tumor marker for GC and CRC, and serum NSE detection could be used for GC and CRC auxiliary diagnosis. Besides, the combined detection of the tumor markers NSE, CEA, CA19-9, and CA242 is of great significance in the diagnosis of GC and CRC.

## Acknowledgments

Thanks to Dr. Sun from Jilin University for providing statistical data analysis

Competing interests.

## Author contributions

Hai Luo and Kexin Shen participated in the design of the experiment and the analysis of all clinical data as well as the revision of the paper. Hongyan Sun participated in the collection and analysis of pathological data. Ruiqi Li participated in the collection and analysis of serum markers. Zeming Wang participated in the collection and analysis of imaging data. Zhongshi Xie participated in data analysis and thesis revision. All authors read and approved the final manuscript.
